# Correlation between Tregs and ICOS-induced M2 macrophages polarization in colorectal cancer progression

**DOI:** 10.3389/fonc.2024.1373820

**Published:** 2024-07-22

**Authors:** Jiaxin Xu, Yu Gao, Yuting Ding, Yunpeng Feng, Jie Chen, Shenshen Zhang, Xiaoyu Song, Shifeng Qiao

**Affiliations:** ^1^ The Second Ward of Colorectal Surgery, The First Affiliated Hospital of Jinzhou Medical University, Jinzhou, Liaoning, China; ^2^ Computer Teaching and Research Section, Jinzhou Medical University, Jinzhou, Liaoning, China; ^3^ Department of Ultrasound, Anshan Central Hospital, Anshan, Liaoning, China

**Keywords:** M2 Macrophages, Tregs, ICOS, colorectal cancer, ICOS

## Abstract

**Objective:**

To explore the mechanism by which Tregs promote the progression of colorectal cancer by inducing tumor-associated macrophages to polarize into M2 type via ICOS.

**Methods:**

Postoperative pathological tissues and clinical pathological data of 268 colorectal cancer patients who underwent initial surgery were collected. Immunohistochemistry (IHC) was used to detect the expression levels of ICOS, CD163 (a marker for M2 macrophages), and Foxp3 (a marker for Tregs) in cancerous, adjacent non-tumorous, and normal tissues. The relationship of ICOS, M2 macrophages, and Tregs in CRC with clinical pathological characteristics and pre-surgical tumor markers (such as CEA and CA199) was explored.

**Results:**

The expression levels of M2 macrophages and Tregs increased with tumor progression, while ICOS expression showed a decreasing trend. Compared to adjacent and normal tissues, the expression levels of ICOS, M2 macrophages, and Tregs were higher in CRC tissues. The expression levels of M2 macrophages and Tregs were significantly positively correlated with tumor markers, while ICOS expression was significantly negatively correlated.

**Conclusion:**

Tumor-associated m2 macrophages induced by Tregs and ICOS participate in the dynamic balance of the colorectal cancer tumor microenvironment, and their interaction affects colorectal carcinogenesis and progression. High levels of ICOS are associated with better long-term survival rates.

## 1 Introduction

According to the International Agency for Research on Cancer (IARC), colorectal cancer (CRC) ranks third in global incidence and has risen to the second leading cause of cancer-related deaths (1). Lifestyle changes have shifted the trend of CRC towards younger age groups and more advanced stages (2). The tumor microenvironment (TME), comprising cancer cells and surrounding non-cancerous cells, including immune cells, stromal cells, and vascular cells, plays a key role in tumor growth and progression (3). M2 macrophages in this environment support tumor growth, angiogenesis, and immune suppression (4). CD163 is identified as a specific marker for M2 macrophages (5). Regulatory T cells (Tregs) maintain tissue homeostasis through immunosuppression but can promote tumor evasion in TME (6). Foxp3, a specific marker for Tregs, plays a critical role in tumor immunity and proliferation (7–9). Inducible T-cell co-stimulator (ICOS), part of the B7-CD28 immunoglobulin superfamily, has a dual role in mediating T cell regulation (10). Initially discovered in human tonsil studies, ICOS acts via its T cell co-stimulator ligand (ICOSL) (11). The decline in co-stimulatory molecules and ligands correlates with lymph node metastasis and tumor invasion in CRC progression (12). ICOS/ICOS-L signaling can lead to the accumulation of Tregs within tumors, influencing the differentiation of CD4 T cells into Tregs (13, 14). Soluble recombinant forms of ICOS (ICOS-CH3) have been found to inhibit M2 macrophage polarization through ICOSL mediation (15). With CRC progression, ICOS expression tends to decrease (16). This study hypothesizes that host anti-tumor immune dysfunction may be due to excessive suppression of the ICOS/ICOSL pathway. Tregs might promote CRC progression by suppressing ICOS expression, inducing polarization of tumor-associated macrophages to the M2 type. The study analyzes ICOS in clinical pathological characteristics through TCGA database and investigates the expression of M2 macrophages, Tregs, and ICOS protein in 268 colorectal tissues using IHC. This research aims to understand the mechanisms of CRC progression and find new breakthroughs in CRC diagnosis and treatment.

## 2 Materials and methods

### 2.1 Clinical data

This study involved 268 colorectal cancer patients who underwent radical surgery in the Second Ward of Colorectal Surgery at The First Affiliated Hospital of Jinzhou Medical University from January 2021 to June 2023. Patient clinical characteristics are detailed in **Table 1**.

**Table 1:** Clinical information statistics.

Variables		N=268	Percent (%)
Age			
	<65	121	45.15
	≥65	147	54.85
Gender			
	Male	164	61.19
	Female	104	38.81
TNM staging			
	I	39	14.55
	II	102	38.06
	III	88	32.84
	IV	39	14.55
Anatomic tumour region			
	Rectum	147	54.85
	Left Colon	62	23.13
	Right Colon	59	22.02

Postoperative pathological tissue samples were categorized into cancerous, adjacent non-tumorous, and normal tissues. These three types of tissues were obtained from cancer tissues, 5cm away from cancer tissues, and 10cm away from cancer tissues of the same patient, respectively. Inclusion criteria were: (1) written informed consent obtained prior to enrollment; (2) primary colorectal cancer; (3) initial diagnosis and surgery at our hospital. Exclusion criteria: (1) chemotherapy or radiotherapy prior to enrollment; (2) synchronous primary tumors or other colorectal cancers; (3) hereditary colorectal cancers. This study was approved by the Ethics Committee of the First Affiliated Hospital of Jinzhou Medical University. All participants signed informed consent before enrollment.

### 2.2 Experimental methods

Immunohistochemistry (IHC): Samples were fixed in formalin, embedded in paraffin, and sectioned at 4 μm thickness. After deparaffinization and hydration, antigen retrieval was performed using EDTA solution (pH=9.0). Endogenous peroxidase blocking and nonspecific staining blocking were performed. Tissue sections were incubated with primary antibodies at 4°C overnight and then with peroxidase-labeled secondary antibodies. Diaminobenzidine (DAB) was used for staining, followed by counterstaining with hematoxylin. Antibodies used included anti-CD163 (1:500, ab182422, Abcam, Cambridge, UK), anti-Foxp3 (1:500, ab20034, Abcam, Cambridge, UK), and anti-ICOS (1:500, ab224644, Abcam, Cambridge, UK) monoclonal antibodies.

### 2.3 Staining result evaluation

CD163+ staining indicates M2 macrophages in the cell membrane or cytoplasm. Foxp3+ staining denotes Tregs in cell nuclei. ICOS+ staining appears in colorectal cell membranes or cytoplasm. Positive control for CD163 and Foxp3 staining was set, while ICOS staining in tonsil tissue sections served as a positive control. Negative control was PBS for all three. Image J was used to determine positive cells, evaluated by two pathologists through a double-blind method.For anti-CD163 and anti-Foxp3 staining, 5 random high power fields (400X) were evaluated to calculate an average number of positive cells. The integer of this average was taken as the final score. The median score of all samples was set as the cut-off, with scores greater than or equal to the median classified as high expression, and scores below as low expression. For ICOS staining, the average staining index across 5 high power fields was calculated for each slide as the final score. Staining index = percentage of positive cells × staining intensity (positive cell percentage was defined as 0: 0–10%; 1: 11–25%; 2: 26–50%; 3: 51–75%; 4: 76–100%; staining intensity was 0: no staining; 1: weak staining; 2: moderate staining, but weaker than tonsil; 3: strong staining, equal to or stronger than tonsil). Staining index ≥6 indicates high expression, <6 indicates low expression.

### 2.4 Statistical analysis

SPSS 26.0 was used for statistical analysis. Categorical variables were presented as number (percentage), and continuous variables were compared using independent sample T-tests or Mann-Whitney tests. Chi-square tests were used for categorical variables. All P-values were two-sided, with *P* < 0.05 considered statistically significant. GraphPad Prism 9.0 was used for graphing.

## 3 Results

### 3.1 Relationship between ICOS expression and colorectal cancer (CRC) in bioinformatics analysis

**Figure 1:**
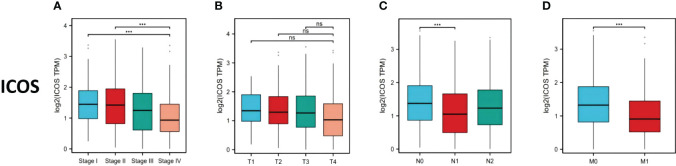
ICOS expression in colorectal cancer patients with different TNM stages. Data were obtained from the TCGA. **(A)** Changes in ICOS expression with CRC TNM stage. **(B)** No significant correlation between ICOS expression and depth of tumor invasion. **(C)** Significant negative correlation between ICOS expression and lymph node metastasis (NO indicates no metastasis, N1 indicates 1-3 regional lymph node metastases, and N2 indicates ≥4 regional lymph node metastases). **(D)** Significant negative correlation between ICOS expression and distant metastasis. Statistical test method: ANOVA, n=669. "***" means less than equal to 0.001; "ns" means greater than 0.05.

**Figure 2:**
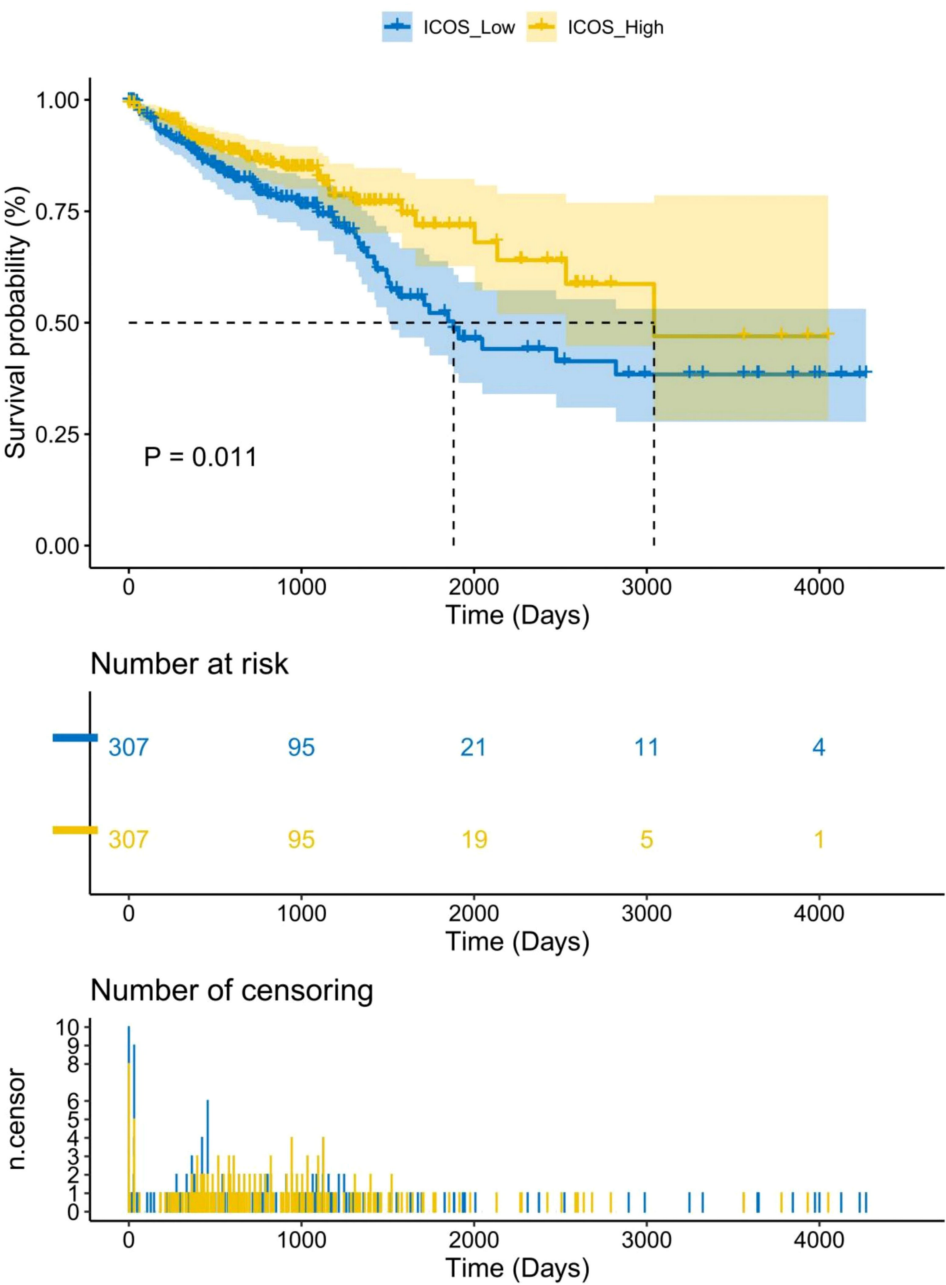
Association between ICOS expression and long-term survival rate of CRC patients. Data were obtained from the TCGA. There is a significant correlation between high expression of ICOS and survival time (*p* = 0.011). The number of long-term risks and censoring for low expression of ICOS is higher than that for high expression of ICOS.Statistical test method: Log-Rank test, n=669.

669 CRC patients were analyzed by TCGA revealed that ICOS expression gradually decreased with the progression of CRC TNM staging (**Figure 1A**). However, there was no correlation between ICOS expression and invasion depth (**Figure 1B**). Lymph node metastasis (**Figure 1C**) and distant metastasis (**Figure 1D**) showed significant correlation with ICOS expression. High expression of ICOS was significantly correlated with long-term survival rates (**Figure 2**).

### 3.2 Expression of CD163, Foxp3, and ICOS in different tissues

**Figure 3:**
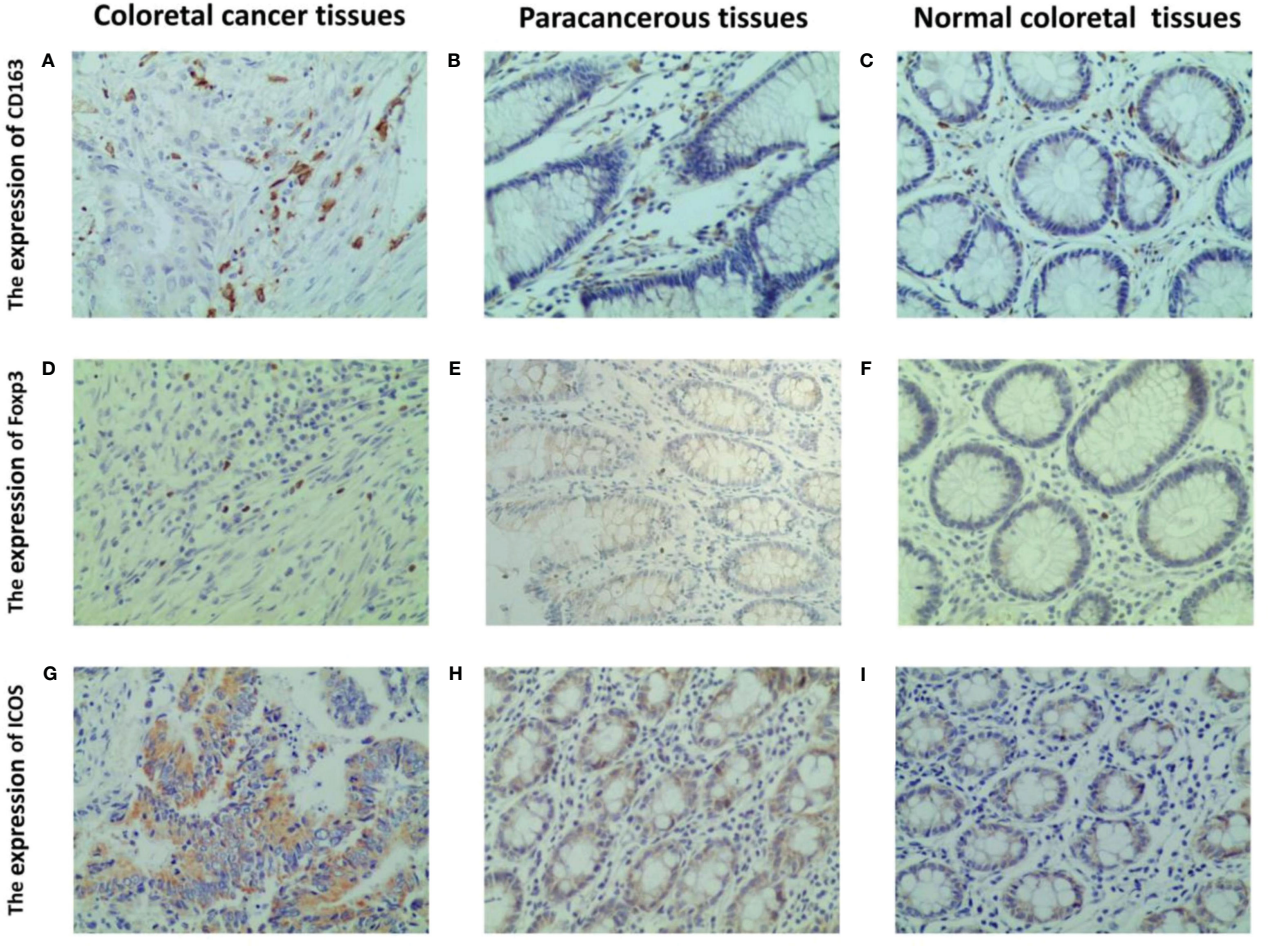
Representative IHC staining of CD163, foxp3 and ICOS in CRC tissues, paracancerous and normal tissues (400 × field). The cell membrane and cytoplasm of M2 macrophages are stained brown **(A–C)**. The nucleus of Tregs are stained brown **(D–F)**. The ICOS protein induces a brown coloration of the cellular membrane and cytoplasm **(G–I)**.

Immunohistochemistry results indicated that CD163 was primarily expressed on the cell membrane and cytoplasm of M2 macrophages, appearing in a brownish-yellow color (**Figure 3A**), and was weaker in peritumoral and normal tissues (**Figures 3B, C**). Foxp3 was predominantly expressed in brownish-yellow granules in the nuclei of Tregs, distributed throughout the cancer tissue in CRC patients (**Figure 3D**). In peritumoral and normal tissues, Foxp3 staining was weaker and localized (**Figures 3E, F**). ICOS was primarily expressed in the cell membrane and cytoplasm of colorectal cells, with stronger staining intensity in cancerous tissue (**Figure 3G**) than in peritumoral (**Figure 3H**) and normal tissues (**Figure 3I**).

## 3.3 Differences in CD163, Foxp3, and ICOS expression in different tissues

**Figure 4:**
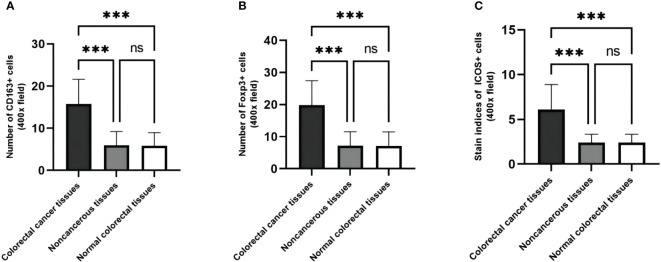
The expression levels of CD163, Foxp3 and ICOS are different in cancer, paracancerous and normal tissues. **(A)** The number of CD163 in CRC tissues, their matched paracancerous and normal tissues. **(B)** The number of Foxp3 in CRC tissues, their matched paracancerous and normal tissues. **(C)** The staining indices of ICOS in CRC tissues, their matched paracancerous and normal tissues. Statistical test method: independent sample t-test, n=268. "***" means less than equal to 0.001; "ns" means greater than 0.05.

When comparing the expression differences of CD163, Foxp3, and ICOS in cancerous, peritumoral, and normal tissues, it was found that their expressions varied among these tissues. Compared to peritumoral and normal tissues, the expression of CD163 (t=26.85, *p <*0.001; t=27.15, *p <*0.001), Foxp3 (t=25.70, *p <*0.001; t=25.76, *p <*0.001), and ICOS (t=24.21, *p <*0.001; t=24.22, *p <*0.001) increased in CRC tissues. There was no statistical difference in the expression of these three proteins between peritumoral and normal tissues (t=0.3056, *p >*0.99; t=0.0606, *p >*0.99; t=0.0097, *p >*0.99) (**Figure 4**).

### 3.4 Relationship between CD163, Foxp3, and ICOS and clinical pathological features of CRC

As shown in **Table 2**, in CRC tissues, CD163 was related to tumor TNM stage (χ2 = 23.974, *p <*0.01), vascular cancer emboli (χ2 = 6.201, *p* =0.010), neural invasion (χ2 = 4.328, *p* =0.029), lymph node metastasis (χ2 = 15.663, *p* =0.002), and distant metastasis (χ2 = 4.273, *p* =0.029). Foxp3 was significantly associated with tumor TNM stage (χ2 = 22.025, *p <*0.01), vascular cancer emboli (χ2 = 5.089, *p* =0.022), neural invasion (χ2 = 5.289, *p* =0.018), lymph node metastasis (χ2 = 15.121, *p* =0.003), and distant metastasis (χ2 = 6.759, *p* =0.007). ICOS was related to tumor TNM stage (χ2 = 18.316, *p <*0.01), vascular cancer emboli (χ2 = 5.050, *p* =0.025), neural invasion (χ2 = 6.305, *p* =0.013), lymph node metastasis (χ2 = 9.287, *p* =0.008), and distant metastasis (χ2 = 7.564, *p* =0.006).

**Table 2:** Clinical and pathological information statistics of CD163, Foxp 3 and ICOS.

Clinicopathological Characteristics		n(%)=268	CD163				FOXP3				ICOS			
			low	high	χ2	p-value	low	high	χ2	p-value	low	high	χ2	p-value
Age (years)					0.199	0.657			0.202	0.655			0.207	0.650
	<65	121(45.1)	51	70			56	65			61	60		
	≥65	147(54.9)	58	89			63	84			70	77		
Gender					0.006	0.940			0.809	0.370			0.085	0.771
	Male	164(61.2)	67	97			76	88			79	85		
	Female	104(38.8)	42	62			43	61			52	52		
Anatomic tumour region					2.004	0.671			1.109	0.508			1.639	0.998
	Rectum	147(54.9)	56	91			62	85			74	73		
	Left colon	65(23.1)	30	35			30	35			26	39		
	Right colon	59(22.0)	23	36			27	32			31	28		
Tumour maximum diameter (cm)					0.032	0.859			1.139	0.288			0.247	0.621
	<5cm	131(48.9)	54	77			62	69			62	69		
	≥5cm	137(51.1)	55	82			57	80			69	68		
Differentiation degree					1.020	0.497			0.933	0.394			0.124	0.864
	Low	29(10.8)	12	17			14	15			15	14		
	Moderate	219(81.7)	91	128			98	121			106	113		
	High	20(7.5)	6	14			7	13			10	10		
TNM staging					23.974	0.000			22.025	0.000			18.316	0.000
	I	39(14.6)	21	18			22	17			15	24		
	II	102(38.0)	56	46			60	42			37	65		
	III	88(32.8)	22	66			28	60			52	36		
	IV	39(14.6)	10	29			9	30			27	12		
Depth of infiltration					10.271	0.915			4.898	0.866			5.612	0.606
	Infiltrate submucosa	11(4.1)	6	5			6	5			4	7		
	Infiltrate muscular	35(13.0)	18	17			19	16			13	22		
	Infiltrate the subserosal layer	147(54.9)	47	100			57	90			81	66		
	Penetrating visceral peritoneum	75(28.0)	38	37			37	38			33	42		
Vascular invasion					6.201	0.010			5.089	0.022			5.050	0.025
	Absent	189(70.5)	86	103			93	97			84	105		
	Present	79(29.5)	23	56			26	53			47	32		
Nerve invasion					4.328	0.029			5.289	0.018			6.305	0.013
	Absent	226(84.3)	98	128			107	119			103	123		
	Present	42(15.7)	11	31			28	14			28	14		
Lymph node metastases					15.663	0.002			15.121	0.003			9.287	0.008
	N0	154(57.5)	78	76			84	70			63	91		
	N1	81(30.2)	20	61			23	58			49	32		
	N2	33(12.3)	11	22			12	21			19	14		
Distant metastases					4.273	0.029			6.759	0.007			7.564	0.006
	Absent	229(85.4)	99	130			110	119			104	125		
	Present	39(14.6)	10	29			9	30			27	12		

### 3.5 Correlation between CD163, Foxp3, and ICOS and TNM staging of CRC

**Figure 5:**
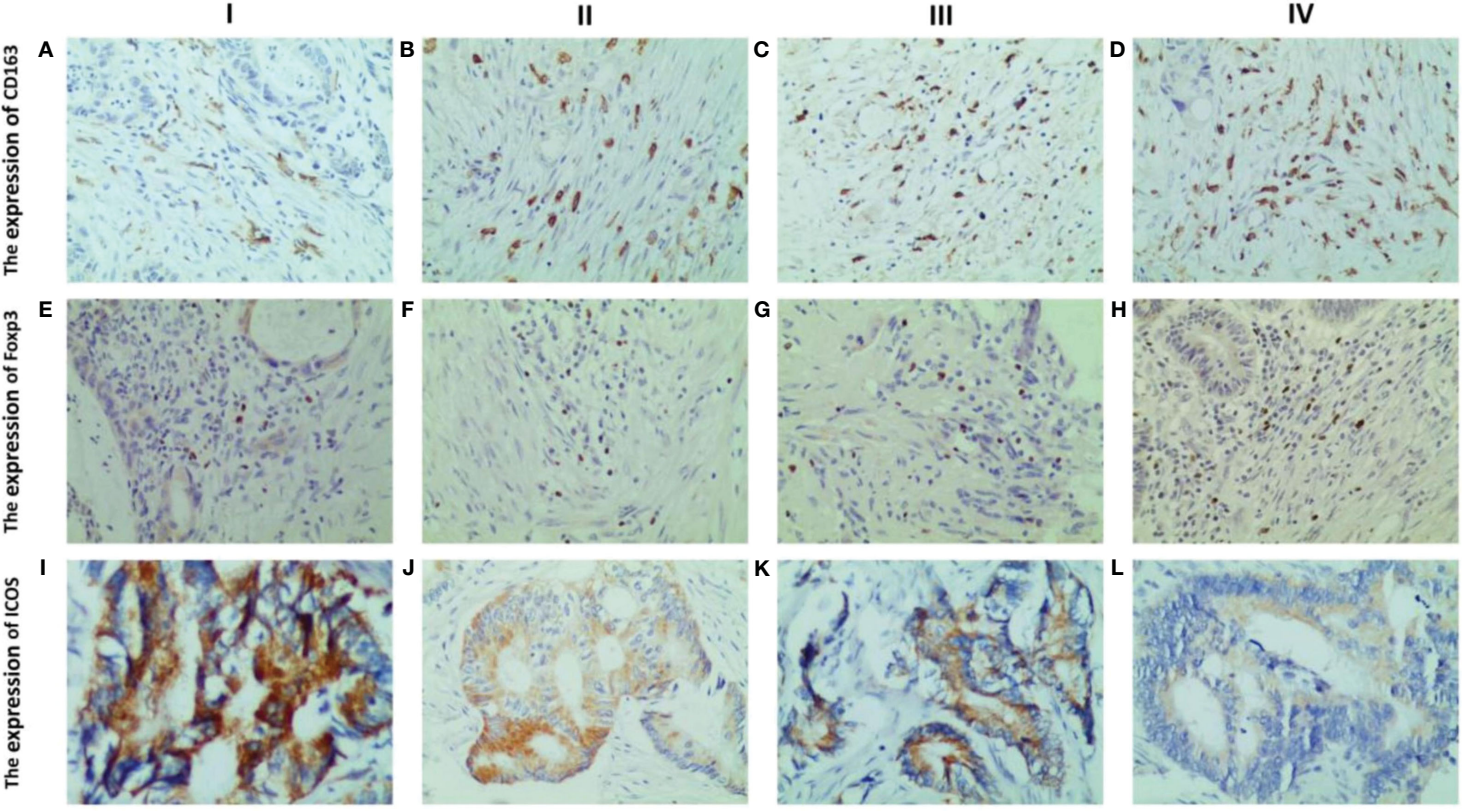
Representative IHC staining of CD163, Foxp3 and ICOS in different tumour TNM staging (400× field). The expression of CD163 in CRC at stages I, II, III and IV **(A–D)**. The expression of Foxp3 in CRC at stages I, II, III and IV **(E–H)**. The expression levels of ICOS in CRC at stages I, II, III and IV **(I–L)**. The expression levels of the three were significantly different in the different TNM stages of CRC patients. With increasing TNM stage, CD163 and Foxp3 expression were significantly increased, while ICOS expression decreased significantly.

Analysis revealed a close relationship between the expression of CD163, Foxp3, and ICOS in CRC tissues and tumor TNM staging. It was found that the expression levels of these three proteins varied in different TNM stages of CRC patients (**Figure 5**).

**Figure 6:**
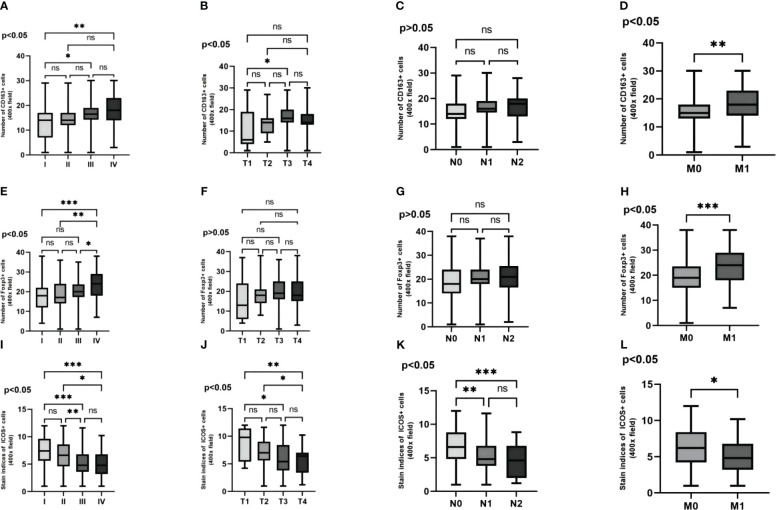
The variability of CD163, Foxp3 and ICOS in the pathological features are examined. The variability of three proteins in the TNM stagings of CRC patients' cancer tissue are analyzed **(A, E, I)**. The variability of these three proteins in the depth of infiltration in CRC patients' cancer tissue are investigated **(B, F, J)**. The variability of these three proteins in the lymph node metastasis in CRC patients' cancer tissue are explored **(C, G, K)**. The variability of these three proteins in the distant metastasis in CRC patients' cancer tissue are evaluated **(D, H, L)**. Statistical test method: ANOVA and independent samples' t-test, n=268. “*” means less than equal to 0.05 but greater than 0.02; “**” means less than equal to 0.02 but greater than 0.001; “***” means less than equal to 0.001; "ns" means more than 0.05.

By comparing the expression levels of these three proteins in TNM stages of CRC patients, it was observed that with the increase in tumor TNM stage, invasion depth, lymph node metastasis, and distant metastasis, the expression levels of CD163 and Foxp3 significantly increased in TNM stages and distant metastasis (**Figures 6A, D, E, H**). And the expression levels of CD163 significantly increased in tumor infiltration depth (**Figure 6B**). However, the expression of ICOS significantly decreased in TNM stages, invasion depth, lymph node metastasis, and distant metastasis (**Figures 6I–L**). The expression level of CD163 did not show statistical significance in terms of lymph node metastasis. Similarly, the expression level of Foxp3 also lacked statistical significance in both infiltration depth and lymph node metastasis (*p >*0.05) (**Figures 6C, F, G**).

### 3.6 Correlation of M2 macrophages, Tregs, and ICOS in CRC

**Figure 7:**
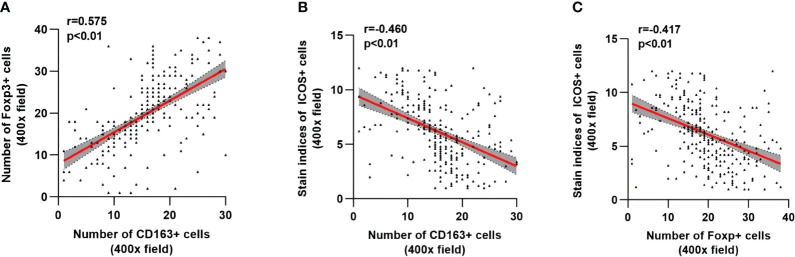
Correlation analysis among CD163, Foxp3 and ICOS in CRC. The linear correlation model is shown with a red line. Corresponding 95% confidence intervals (CI) are shown with the gray area. **(A)** Correlation between the number of CD163^+^ cells and Foxp3^+^ cells. **(B)** Correlation between the number of CD163^+^ cells and the staining indices of ICOS^+^ cells. **(C)** Correlation between the number of Foxp3+ cells and the staining indices of ICOS cells. Statistical test method: Spearman correlation analysis.

As shown in **Figure 7**, the number of M2 macrophages was significantly positively correlated with the number of Tregs (r=0.575, *p <*0.01) (**Figure 7A**), while the number of M2 macrophages and Tregs was significantly negatively correlated with the ICOS staining index (r=-0.460, *p <*0.01; r=-0.417, *p <*0.01) (**Figures 7B, C**).

### 3.7 Correlation between the number of M2 macrophages, Tregs, ICOS staining index, and tumor markers in CRC

As shown in **Table 3**, Spearman correlation analysis was used to determine the correlation between the number of M2 macrophages and Tregs, ICOS staining index, and preoperative CEA, CA19–9, and CA72–4 in CRC. The number of M2 macrophages was significantly positively correlated with preoperative CEA (r=0.190, *p* =0.002), but had no significant correlation with preoperative CA19–9 and CA72–4 (r=0.116, *p* =0.058; r=0.037, *p* =0.550). The number of Tregs was significantly positively correlated with preoperative CEA and CA19–9 (r=0.205, *p* =0.001; r=0.183, *p* =0.003), but had no significant correlation with preoperative CA72–4 (r=0.008, *p* =0.892). The ICOS staining index was significantly negative correlated with preoperative CEA and CA19–9 (r=-0.176, *p* =0.004; r=-0.158, *p* =0.010), but had no significant correlation with preoperative CA72–4 (r=-0.056, *p* =0.362).

**Table 3:** Correlation analysis of the number of M2 macrophages and tregs and stain Indices of ICOS Cells with Preoperative CEA, CA19-9 and CA72-4.

Tumour Markers	The Mean Number of M2 Macrophages		The Mean Number of Tregs		Stain Indices of ICOS+ Cells	
	r	*p*-value	r	*p*-value	r	*p*-value
Preoperative CEA (μg/L)	0.190	0.002	0.205	0.001	-0.176	0.004
Preoperative CA19-9 (U/mL)	0.116	0.058	0.183	0.003	-0.158	0.010
Preoperative CA72-4 (U/mL)	0.037	0.550	0.008	0.892	-0.056	0.362

## 4 Discussion

This study, involving immunohistochemical (IHC) staining of cancerous, peritumoral, and normal tissues from 268 colorectal cancer (CRC) patients, has for the first time discovered a correlation between ICOS, M2 macrophages, and regulatory T cells (Tregs) in CRC. As CRC progresses, ICOS exhibits a significant negative correlation with M2 macrophages and Tregs. The expression levels of M2 macrophages and Tregs increase with tumor progression, whereas ICOS expression decreases. This trend may be associated with the ICOS/ICOSL signaling pathway. All three proteins showed higher expression in cancerous tissues compared to peritumoral and normal tissues. Bioinformatics analysis indicated that high ICOS expression significantly impacts long-term survival rates.

M2 macrophages and Tregs are vital components of the tumor microenvironment (TME) and play a crucial role in the development and progression of malignancies. Previous studies have found a close relationship between M2 macrophages and Tregs in various cancers, including gastric, colorectal, ovarian epithelial, and prostate cancers (17–20). M2 macrophages, as the primary expression form of tumor-associated macrophages, promote Th2 cell differentiation through the induction of IL-4, IL-13, and other cytokines and secrete IL-10, TGF-β to suppress inflammatory cytokines (4). Tumor-associated M2 macrophages play a significant role in tumor progression and immune escape by secreting anti-inflammatory factors and activating related signaling pathways (21). Studies by Gigliotti CL and others have shown that ICOS-CH3, by activating ICOSL, differentially regulates human M1 and M2 macrophages under various polarization conditions. ICOS-CH3 inhibits M2 macrophage polarization, thus reducing cytokine secretion and cell migration (15). Chang SR’s findings in advanced head and neck squamous cell carcinoma (HNSCC) and oral squamous cell carcinoma (OSCC) indicate increased M2 macrophage expression and decreased ICOS expression (22). Our study corroborates these findings.

Tregs, as a critical subset of functionally suppressive T cells, play a vital role in immune self-tolerance (6). They can suppress the function of effector T cells either through direct cell-to-cell contact or by secreting immunosuppressive factors (9). Research by Liyanage has shown that in pancreatic or breast cancer, the proportion of Tregs in tumor-infiltrating lymphocytes (TILs) and peripheral blood increases. Tregs suppress the proliferation of CD4+ and CD8+ T cells, reducing anti-tumor immune responses and are closely related to tumor prognosis (23). Studies have found that ICOS, by triggering ICOSL, promotes the differentiation of Tregs and Th-17 cells, regulating T cell activation in lymphoid organs and T cell function at inflammatory sites (24, 25). Mesturini and others discovered that ICOS in the TME inhibits the differentiation of naïve T cells into Tregs through its soluble ligand form, thereby inhibiting tumor progression (26). We hypothesize that the increase in Tregs could inversely inhibit the ICOS/ICOSL signaling pathway, further suppressing effector T cell immune responses against the tumor and promoting CRC development.

ICOS, a co-stimulatory receptor homologous to the stimulatory receptor CD28 and inhibitory receptor CTLA-4, plays a dual role in regulating T cell function across different tumors (10, 27). Programmed cell death-1 ligand 1 (PD-L1) leads to upregulation of M2 macrophages in the TME through activation of signal transducer and activation of transcription 3 (STAT 3)/nuclear factor kappa-B (NF- κ B) signaling pathway, leading to immunosuppression and EMT in CRC (28). The FDA-approved ipilimumab, a CTLA-4 blocking antibody, has shown success in various malignancies by targeting the PD-1 pathway, highlighting the significance of the B7-CD28 family in tumor immunotherapy (29). ICOS-mediated ICOSL triggering drives a “reverse signal” that inhibits migration and cytokine secretion in endothelial cells (ECs), dendritic cells (DCs), and tumor cells (30–33). Additionally, ICOSL triggering promotes antigen cross-presentation in dendritic cells and inhibits osteoclast (OC) differentiation and function (34). In tumor cells, high-level ICOSL expression activated by ICOS significantly reduces tumor cell metastasis, indicating that the inhibitory effect of ICOS-mediated ICOSL triggering surpasses the promoting effect of OPN-mediated ICOSL triggering. Furthermore, ICOS-Fc treatment increases effector T cells and reduces regulatory T cells, inhibiting tumor cell metastasis (35). In OSCC and HNSCC, higher ICOS or lower CD276/ICOS is a good prognostic marker for patient survival and lymph node metastasis (22). This is consistent with our findings where ICOS expression decreases with CRC progression, and high ICOS expression is significantly correlated with long-term survival (16).

The interaction between M2 macrophages and Tregs can promote CRC progression (18). In the TME, inhibiting the ICOS/ICOSL signaling pathway promotes the differentiation of naive T cells into Tregs (14). High levels of Tregs might induce tumor-associated macrophages to polarize towards the M2 type by inhibiting ICOS expression. Tumor markers have been widely used in CRC research, with their specificity indices playing a crucial role in early cancer screening and postoperative recovery (36). Preoperative CEA has been found to be significantly positively correlated with the expression levels of M2-type macrophages and Tregs in CRC, closely related to CRC lymph node metastasis, consistent with our research findings (18). Dianzani C and others found that ICOS-Fc treatment inhibited tumor cell migration to the lungs in mice, increasing IL-17A and RAR-related orphan receptor C (RORc) expression while reducing IL-10 and Foxp3 expression. ICOS-Fc inhibited tumor epithelial-mesenchymal transition (EMT) and migration *in vitro* and metastasis *in vivo* (32). M2 macrophages and Tregs in CRC have a synergistic effect, with the inhibition of the ICOS/ICOSL signaling pathway likely playing a key role. Although ICOS’s role in tumor immunology has been extensively studied, its specific role and mechanism in CRC remain unclear. Particularly, the role of ICOS in regulating Tregs and affecting M2-type macrophage polarization requires further exploration. This study aims to fill this research gap, with future investigations focusing on how Tregs promote CRC development by inducing M2-type macrophage polarization through ICOS. The dual role of ICOS in different malignancies is clear, but its positive prognostic significance in CRC is undeniable.

However, this study has limitations. As a cross-sectional study, we cannot infer causality and need more longitudinal studies to verify our results. Secondly, the participants’ origin from China may limit the generalizability of our findings. Lastly, immunohistochemistry has limitations in repeatability and accuracy compared to more advanced methods.

## 5 Conclusion

The expression levels of M2 macrophages and Tregs increased with tumor progression, while ICOS expression showed a decreasing trend. Tregs may promote CRC tumor progression by downregulating ICOS and inhibiting the ICOS/ICOSL signaling pathway, inducing M2-type macrophage polarization. M2-type macrophages, Tregs, and the ICOS/ICOSL signaling pathway could become new potential targets for immune therapy in CRC, holding significant clinical value for the diagnosis, treatment, and prognosis of CRC patients.

## Data availability statement

The data that support the findings of this study are available on request from the corresponding author.

## Ethics statement

The study was conducted in accordance with the Ethics Committee of the First Affiliated Hospital of Jinzhou Medical University (KYLL 202463) and the 1964 Helsinki Declaration. Written informed consent obtained from all participants included in the study.

## Author contributions

JX: Conceptualization, Data curation, Formal analysis, Funding acquisition, Investigation, Methodology, Project administration, Resources, Software, Supervision, Validation, Visualization, Writing – original draft, Writing – review & editing. YG: Conceptualization, Data curation, Formal analysis, Funding acquisition, Investigation, Methodology, Project administration, Resources, Software, Supervision, Validation, Visualization, Writing – original draft, Writing – review & editing. YD: Writing – review & editing. YF: Writing – review & editing. JC: Writing – review & editing. SZ: Writing – review & editing. XS: Writing – review & editing. SQ: Writing – original draft, Writing – review & editing, Conceptualization, Data curation, Formal analysis, Funding acquisition, Investigation, Methodology, Project administration, Resources, Software, Supervision, Validation, Visualization.
